# Pathogenic Variant Filtering for Mitochondrial Genome Haplotype Reporting

**DOI:** 10.3390/genes11101140

**Published:** 2020-09-28

**Authors:** Charla Marshall, Kimberly Sturk-Andreaggi, Joseph D. Ring, Arne Dür, Walther Parson

**Affiliations:** 1 Armed Forces Medical Examiner System’s Armed Forces DNA Identification Laboratory (AFMES-AFDIL), Dover Air Force Base, DE 19902, USA; kimberly.s.andreaggi.ctr@mail.mil (K.S.-A.); joseph.d.ring2.ctr@mail.mil (J.D.R.); 2 SNA International, Contractor Supporting the AFMES-AFDIL, Alexandria, VA 22314, USA; 3 Forensic Science Program, The Pennsylvania State University, University Park, PA 16802, USA; 4 Department of Immunology, Genetics and Pathology, Uppsala University, 75185 Uppsala, Sweden; 5 Institute of Mathematics, University of Innsbruck, 6020 Innsbruck, Austria; arne.duer@uibk.ac.at; 6 Institute of Legal Medicine, Medical University of Innsbruck, 6020 Innsbruck, Austria

**Keywords:** mitochondrial genome, mitochondrial DNA, coding region, variant filtering, pathogenic variants, haplotype, haplogroup, population statistics, random match probability, EMPOP

## Abstract

Given the enhanced discriminatory power of the mitochondrial DNA (mtDNA) genome (mitogenome) over the commonly sequenced control region (CR) portion, the scientific merit of mitogenome sequencing is generally accepted. However, many laboratories remain beholden to CR sequencing due to privacy policies and legal requirements restricting the use of disease information or coding region (codR) information. In this report, we present an approach to obviate the reporting of sensitive codR data in forensic haplotypes. We consulted the MitoMap database to identify 92 mtDNA codR variants with confirmed pathogenicity. We determined the frequencies of these pathogenic variants in literature-quality and forensic-quality databases to be very low, at 1.2% and 0.36%, respectively. The observed effect of pathogenic variant filtering on random match statistics in 2488 forensic-quality mitogenome haplotypes from four populations was nil. We propose that pathogenic variant filtering should be incorporated into variant calling algorithms for mitogenome haplotype reporting to maximize the discriminatory power of the locus while minimizing the reveal of sensitive genetic information.

## 1. Introduction

The field of forensic genetics is poised to adopt complete mitochondrial genome (mitogenome) sequencing for use in routine casework. Given the enhanced discriminatory power of the mitogenome over the commonly sequenced mitochondrial DNA (mtDNA) control region (CR) portion, (e.g., [1,2,3]), combined with the technological capability for massively parallel sequencing (MPS) of highly degraded samples [4,5,6,7], the scientific merit of mitogenome sequencing is generally accepted. However, many laboratories remain beholden to CR sequencing until the use of the entire mitogenome can be accepted in court, as the affiliated legal system(s) in some jurisdictions prohibit coding region (codR) DNA evidence, as it may reveal sensitive genetic information about the DNA donor. As the multicopy organellar genome housed in each cell’s mitochondria, mtDNA codes for genes and RNA molecules that enable mitochondrial function to provide cellular energy in a process called respiration. Pathogenic variants in the mtDNA codR are known, for example, to impair metabolism, cause vision loss and deafness, and result in systemic dysfunction of the brain and nervous system [8]. There are several diseases associated with these pathogenic variants, including LHON (Leber hereditary optic neuropathy), MELAS (mitochondrial encephalomyopathy, lactic acidosis, and stroke-like episodes), and Leigh disease [8]. Even more diseases, such as endometriosis, cyclic vomiting syndrome, and many others, have been shown to be potentially correlated with mtDNA sequence variants, but the causative links are lacking. Since mitogenome sequencing and the forensic reporting of mtDNA haplotypes may reveal disease-related information contained within the codR that may encroach upon privacy rights and is generally not needed for forensic identification, pathogenic alleles can be filtered in order to obviate the reporting of sensitive codR information.

## 2. Materials and Methods

**Figure 1:**
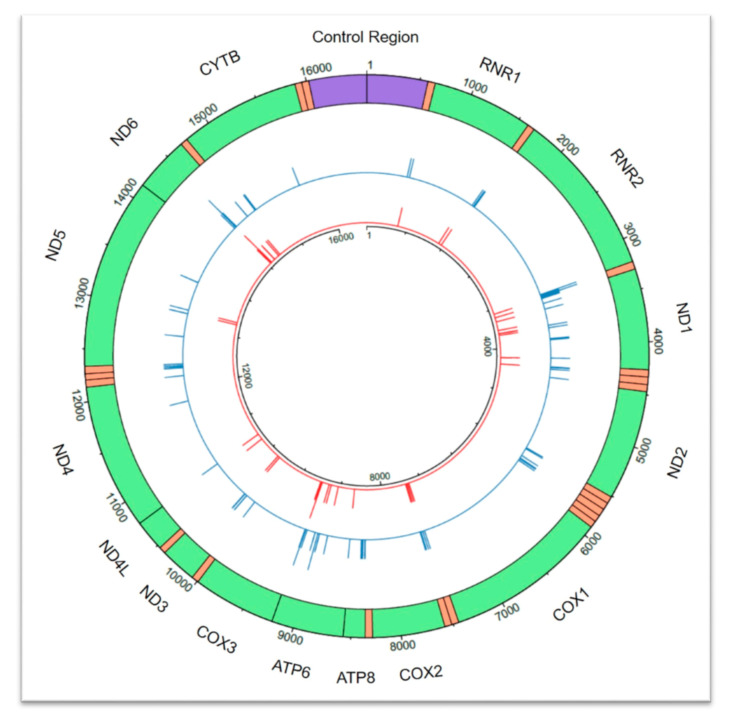
Circular depiction of the 92 confirmed pathogenic variants affecting 92 coding region (codR) positions across the mitochondrial genome as described in MitoMap [8]. Homoplasmic state (*n* = 37) pathogenic variants are visible in the inner circle in red, and heteroplasmic state (*n* = 83) pathogenic variants are presented in the outer circle in blue. Pathogenic variants are represented by a line at the corresponding nucleotide position. The height of the line corresponds to the number of pathogenic variants observed at each nucleotide position, up to a maximum of two for both homoplasmic and heteroplasmic states. The control region is shown in purple, transfer RNA (tRNA) regions are orange, and codR genes are green and labeled accordingly with the corresponding gene. RNR1 = 12S RNA; RNR2 = 16S RNA; ND1 = NADH dehydrogenase 1; ND2 = NADH dehydrogenase 2; COX1 = cytochrome c oxidase I; COX2 = cytochrome c oxidase 2; ATP8 = ATP synthase 8; ATP6 = ATP synthase 6; COX3= cytochrome c oxidase 3; ND3 = NADH dehydrogenase 3; ND4L = NADH 4L dehydrogenase; NADH4 = NADH dehydrogenase 4; ND5 = NADH dehydrogenase 5; NADH6 = NADH dehydrogenase 6; CYTB = cytochrome b. This figure was generated using the circlize package v0.4.10 in R [12].

MitoMap is a human mtDNA database that continuously compiles the published literature pertaining to mtDNA variation [8]. We utilized the June 2020 version of MitoMap: Mitochondrial DNA Base Substitution Diseases [9,10] to identify confirmed pathogenic variants in the mtDNA codR. The pathogenicity status of each variant is classified in MitoMap as one of the following: confirmed, reported, conflicting reports, or point mutation (non-pathogenic polymorphism). Variants are only confirmed to be pathogenic “if two or more independent laboratories have published reports on the pathogenicity of a mutation. These mutations [with confirmed status] are generally accepted by the mitochondrial research community as being pathogenic” [8]. Those codR variants that are confirmed to be pathogenic are listed in Table 1 and shown graphically in Figure 1 (and further details are provided in Table S1). Many of the pathogenic codR variants are observed in heteroplasmic states, in which individuals have both “normal” (non-pathogenic, e.g., the revised Cambridge Reference Sequence, or rCRS, nucleotide [11]), and deleterious (pathogenic) alleles in their many copies of mtDNA. The pathogenicity of such heteroplasmic variants may be dependent on the quantitative contribution of the pathogenic variant. This manifests as a mixture at the affected position provided that the analytical thresholds defined by the individual laboratory allow for the detection of the minor contributor.

A query of each confirmed pathogenic base and corresponding International Union of Pure and Applied Chemistry (IUPAC) codes to denote heteroplasmic states was performed in EMPOP (EDNAP mtDNA Population Database, https://empop.online) in order to assess the frequency in global populations [13]. EMPOP hosts a dataset of 26,013 full mitogenome sequences downloaded from GenBank and vetted according to criteria specified for EMPOP quality control [13]. This dataset may still contain some errors, as the sequences were not produced following forensic standards (e.g., [14,15]); however, all eye-catching and redundantly-appearing data idiosyncrasies were removed or corrected. This dataset can be utilized to predict and search haplogroup affiliations based on individual variants (see https://empop.online/hg_tree_browser [16]). However, it is not useable to estimate haplotype frequencies for forensic purposes, as most of the haplotypes lack metadata that would be necessary to describe the statistical results.

The effect of pathogenic variant filtering on haplotype frequency estimations was evaluated using forensic-quality mitogenome datasets (Table S2). The datasets are described in [1,2,3,17,18,19,20,21,22,23,24] with the exception of two datasets that were recently submitted to and quality-controlled by EMPOP but are not published yet [25]. The datasets included 2488 haplotypes representing four global populations (West Eurasian *n* = 623, African *n* = 613, East Asian *n* = 630, and Hispanic/Native American *n* = 622). The datasets were imported into the laboratory information systems application (LISA; FTI, Reston, VA) and stored by population for analysis. Each population dataset was then duplicated and any pathogenic variants (homoplasmic or heteroplasmic) were filtered from the haplotypes. Additionally, the deletion at np 3107 was added to the variant filter list in order to remove this artefactual variant from the reported haplotypes [11]. The CRS contained two C nucleotides at nps 3106 and 3107 [26], which was corrected to a single cytosine residue in the rCRS [11]. The 3107N placeholder was kept in the rCRS to maintain the numbering system for all positions downstream of that particular region. Pairwise comparisons were performed in LISA for each population with and without pathogenic variant filtering to obtain the haplotype frequencies, calculate the observed random match probability (RMP) using the sum of squares, and estimate haplotype diversity. The pairwise comparisons excluded indels at common mtDNA regions of length heteroplasmy (i.e., nps 16193, 309, 455, 463, 473, 960, 5899, 8276, and 8285). Analyses were conducted both using discrepant point heteroplasmies as differences between otherwise identical sequences (i.e., literal match mode in EMPOP) and in an inclusive way, i.e., heteroplasmies are disregarded as differences between otherwise identical sequences (pattern match mode in EMPOP; see EMPOP documentation at https://empop.online/use for more details).

## 3. Results

Twenty-nine of the 92 (31.5%) possible confirmed pathogenic variants were found in the EMPOP database out of 26,013 literature-quality haplotypes (Table 2). The relative proportion of haplotypes with a confirmed pathogenic variant was 1.2% (309 out of 26,013) (Table S3). The most frequently observed pathogenic variant was G11778A, which was found in 133 haplotypes (131 as homoplasmic and two as heteroplasmic) for an observed frequency of 0.51%. The G11778A variant is known to be highly recurrent [27] and was associated with 115 different haplogroups in our dataset (Table S3; 110 haplogroups with G11778A and three haplogroups (G2b1a, M5, and U2e1b) with single observations of a combination of G11778A and A1555G and two haplogroups with heteroplasmic G11778A(R)). The second most commonly observed confirmed pathogenic variant was T14484C at 0.22% (55 homoplasmic and one heteroplasmic), followed by A1555G at 0.14%, G3460A at 0.10% (25 homoplasmic and two heteroplasmic), and C14568T at 0.023%. As with G11778A, all four variants were observed in diverse haplogroups. Of note, three of the 309 haplotypes contained two pathogenic variants, and the remaining 306 haplotypes contained only a single pathogenic variant. All three of the haplotypes with two confirmed pathogenic variants exhibited G11778A and A1555G; however, each of these haplotypes was unique and belonged to a different haplogroup. Twenty-four of the 29 observed pathogenic variants were found fewer than five times each in our dataset of 26,013 haplotypes. In fact, approximately half (14) of the observed pathogenic variants were found below 0.01% frequency, i.e., with only one or two observations each. The majority (68.5%) of the 92 confirmed pathogenic variants (*n* = 63) were not observed among the dataset.

As the literature-quality dataset of 26,013 haplotypes may include haplotypes with errors, despite rigorous quality control [13], a separate, forensic-quality EMPOP dataset was used to examine the effect of pathogenic variant filtering on haplotype frequency estimations and RMP. This smaller dataset of 2488 haplotypes represents the highest quality mitogenome sequences from previously (or soon-to-be) published population samples. Four global populations were included: West Eurasian, African, East Asian, and Hispanic/Native American. A total of nine haplotypes were identified with confirmed pathogenic variants in this smaller dataset (Table S4), decreasing the proportion of mitogenomes with a confirmed pathogenic variant to 0.36% when compared to the literature-quality data (1.2%). This discrepancy may be related to the fact that forensic studies typically involve samples from healthy donors, while the data from the literature included diseased individuals. The specific pathogenic variants found in the forensic-quality dataset were A1555G (*n* = 5), G11778A(R) (*n* = 2), G3733A(R) (*n* = 1), and T14484C (*n* = 1). However, three of the five haplotypes with pathogenic variant A1555G were identical and sampled from a population isolate in Tibet [20]. Therefore, these identical haplotypes may represent maternal relatives, thereby inflating the true frequency of the haplotype in the broader East Asian population. Even when considering the identical haplotypes in the 2488-sample dataset, the frequency of confirmed pathogenic variants in random population data is low, at less than one half of one percent. It is also of note that the most common literature-quality pathogenic variant, G11778A, was observed in the forensic-quality dataset, but as a heteroplasmic variant (G11778R) rather than homoplasmic. Furthermore, the next two most common pathogenic variants based on the literature-quality dataset of 26,013 samples were also observed in the forensic-quality population samples (A1555G and T14484C). When the nine observed pathogenic variants were filtered from the four population datasets, there was no change in the number of unique haplotypes, observed RMP, and haplotype diversity (Table 3), regardless of the handling of point heteroplasmies (Table S5). In other words, the effect of removing the pathogenic variants on haplotype match statistics was nil for all four populations.

## 4. Discussion

**Figure 2:**
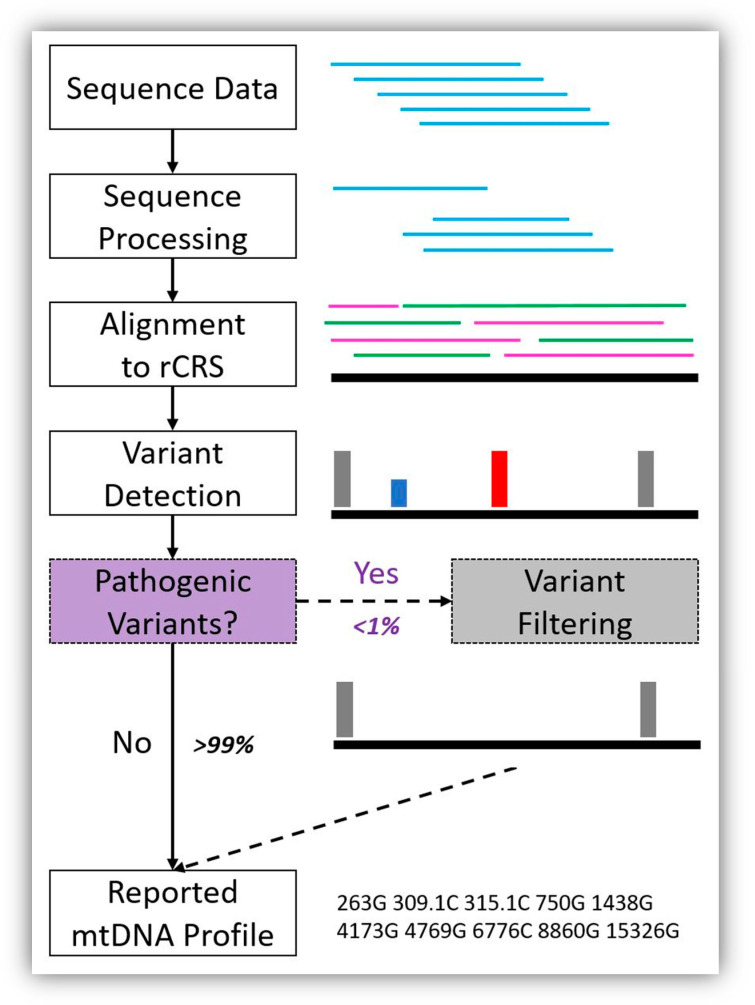
Example bioinformatics workflow showing the addition of pathogenic variant filtering to the analytical process for forensic mitochondrial genome (mitogenome) haplotype reporting. Sequence data are produced, processed, and aligned to the revised Cambridge Reference Sequence (rCRS) [11] for variant detection. Then, pathogenic variant filtering will be automatically performed before the mitochondrial DNA (mtDNA) profile is reported. Here, the pathogenic variants are depicted in red (homoplasmic) and blue (heteroplasmic), and both are filtered. Our results indicate that less than 1% of mtDNA haplotypes will require pathogenic variant filtering and the remaining 99% of mtDNA haplotypes will remain unchanged because they lack pathogenic variants.

We propose that commercial suppliers of mtDNA analysis software take advantage of the pathogenic variant filter list provided in this study that will also be made available in the future as a curated version via the EMPOP webpage (https://empop.online). Laboratories using general bioinformatics software, such as CLC Genomics Workbench or GATK, could implement variant filtering within their analytical pipelines to remove these pathogenic variants prior to data analysis. This would allow forensic users to take advantage of automatically filtered mitogenome variant data; so pathogenic variants (and the 3107 deletion) would not make it into reports that are used to produce and document case-specific data. We provide an example analytical workflow that incorporates pathogenic variant filtering in Figure 2.

Full mitogenome data are considered to be sensitive genetic data due to the potential for revealing phenotypic or disease-related information in the mtDNA codR. At the time of writing, 92 mitochondrial DNA codR variants have been confirmed as pathogenic variants by two or more independent laboratories, as described in the mitochondrial variant database MitoMap. This report demonstrates that the 92 confirmed pathogenic variants affecting 92 unique positions in the mtDNA codR are found at very low frequencies in both literature-quality (*n* = 26,013) and forensic-quality (*n* = 2488) mitogenome datasets, at 1.2% and 0.36%, respectively. Correspondingly, these pathogenic variants can be filtered from forensic mitogenome haplotypes with no observed effect on population statistics including the number of unique haplotypes, observed RMP, and haplotype diversity. Variant filters are frequently utilized in bioinformatics toolkits in other disciplines and these can be implemented in the commonly used forensic mtDNA analysis software programs such as AQME [28], GeneMarker HTS [29], or Converge [6]. By reporting mitogenome haplotypes after filtering confirmed pathogenic variants, forensic laboratories will be able to take advantage of the enhanced discriminatory power of the complete mtDNA locus including the codR variation while minimizing the risk of revealing sensitive genetic information, as previously suggested [30].

## Figures and Tables

**Table 1:** List of the 92 confirmed pathogenic variants described in MitoMap [8], separated by homoplasmic state (*n* = 37) or heteroplasmic state (*n* = 83). Note that there are two multi-nucleotide variants affecting multiple nucleotide positions: the dinucleotide deletion at T9205-A9206 and the sequence inversion causing A3902G C3904A T3905A T3906G C3908T. Conversely, there are five nucleotide positions with two different pathogenic variants each (e.g., T9176C and T9176G). Thus, the 92 confirmed pathogenic variants affected 92 unique coding region (codR) positions. Variants that are confirmed to be pathogenic in both homoplasmic and heteroplasmic states are underlined.

Homoplasmic Variants with Confirmed Pathogenicity	Heteroplasmic Variants with Confirmed Pathogenicity
T616C, C1494T, A1555G, C3303T, G3376A, G3460A, G3635A, G3697A, G3700A, G3733A, C4171A, A4300G, A7445G, C7471CC, G7497A, T7511C, T8528C, T8851C, T8993G, T9035C, T9176C, T9176G, T9185C, T9205del A9206del, T10158C, G10197A, T10663C, G11778A, G13051A, T13094C, G14459A, C14482A, C14482G, T14484C, C14568T, T14674C, T14709C	G583A, T616C, G1606A, A1630G, G1644A, A3243G, A3243T, C3256T, T3258C, A3260G, T3271del, T3271C, A3280G, T3291C, A3302G, C3303T, G3376A, G3460A, G3697A, G3733A, G3890A, A3902G C3904A T3905A T3906G C3908T, C4171A, G4298A, A4300G, G4308A, G4332A, G4450A, G5521A, A5537AT, G5650A, A5690G, G5703A, T5728C, A7445G, C7471CC, G7497A, T7510C, T7511C, T8306C, G8313A, G8340A, A8344G, T8356C, G8363A, T8528C, T8851C, G8969A, T8993C, T8993G, T9035C, A9155G, T9176C, T9176G, T9185C, T10010C, T10158C, T10191C, G10197A, C11777A, G11778A, G12147A, C12258A, G12276A, G12294A, G12315A, G12316A, T12706C, G13042A, T13094C, G13513A, A13514G, G14459A, C14482A, C14482G, T14484C, T14487C, A14495G, T14709C, G14710A, T14849C, T14864C, A15579G

**Table 2:** The 29 confirmed pathogenic variants found in literature-quality mitogenomes (*n* = 26,013) in the EDNAP mtDNA Population Database (EMPOP) [13]. Heteroplasmic states are indicated in parentheses with the corresponding IUPAC code. Pathogenic variants that were observed in both homoplasmic and heteroplasmic states are underlined.

Count of Observations	Observed Frequency (*n* = 26,013)	Confirmed Pathogenic Variants Observed
1	0.004%	T616C, C4171A, A4300G, G5703A(R), A7445G, T9176G, T9185C(Y), T10663C, G12276A(R), G13051A, G13513A(R), G14459A(R), T14484C(Y), T14709C(Y)
2	0.008%	G3460A(R), G3700A, G3733A, C4171A(M), T8851C, G11778A(R), C14482A
3	0.012%	C1494T, T8993G, T9176C, T9185C, G10197A, G14459A
4	0.015%	G3635A, C7471CC, T14674C
6	0.023%	C14568T
25	0.096%	G3460A
37	0.142%	A1555G
55	0.211%	T14484C
131	0.504%	G11778A

**Table 3:** The change in the number of unique haplotypes, observed random match probability (RMP), and haplotype diversity estimates when considering point heteroplasmies (i.e., literal search mode) for forensic-quality mitochondrial genome datasets after filtering confirmed pathogenic variants.

		Original Dataset			Filtered Dataset			
Population	Samples	Unique Haplotypes	RMP	Haplotype Diversity	Pathogenic Variants Filtered	Change in Unique Haplotypes	Change in RMP	Change in Haplotype Diversity
West Eurasian	623	575 (30 shared)	0.21%	0.9995	1	0	0%	0
African	613	597 (15 shared)	0.17%	0.9999	3	0	0%	0
East Asian	630	557 (45 shared)	0.22%	0.9994	4	0	0%	0
Hispanic/Native American	622	568 (43 shared)	0.20%	0.9996	1	0	0%	0

## Supplementary Materials

The following are available online at https://www.mdpi.com/2073-4425/11/10/1140/s1, Table S1. Confirmed pathogenic variants in the mitochondrial genome, as defined by MitoMap r187; Table S2. EMPOP forensic-quality population datasets for the statistical evaluation of the forensic mitogenome pathogenic variant filter; Table S3. Pathogenic variants identified in the literature-quality EMPOP database of 26,013 samples; Table S4. Pathogenic variants observed in high-quality population datasets submitted to EMPOP, representing 2488 samples; Table S5. The change in the number of unique haplotypes, observed random match probability (RMP), and haplotype diversity estimates ignoring point heteroplasmies (i.e., pattern search mode) for forensic-quality mitochondrial genome datasets after filtering.

## References

[1] King J.L., LaRue B.L., Novroski N.M., Stoljarova M., Seo S.B., Zeng X., Warshauer D.H., Davis C.P., Parson W., Sajantila A. (2014). High-Quality and High-Throughput Massively Parallel Sequencing of the Human Mitochondrial Genome using the Illumina MiSeq. Forensic Sci. Int. Genet..

[2] Just R.S., Scheible M.K., Fast S.A., Sturk-Andreaggi K., Röck A.W., Bush J.M., Higginbotham J.L., Peck M.A., Ring J.D., Huber G.E. (2015). Full mtGenome Reference Data: Development and Characterization of 588 Forensic-Quality Haplotypes Representing Three US Populations. Forensic Sci. Int. Genet..

[3] García Ó., Alonso S., Huber N., Bodner M., Parson W. (2020). Forensically Relevant Phylogeographic Evaluation of Mitogenome Variation in the Basque Country. Forensic Sci. Int. Genet..

[4] Parson W., Strobl C., Huber G., Zimmermann B., Gomes S.M., Souto L., Fendt L., Delport R., Langit R., Wootton S. (2013). Evaluation of Next Generation mtGenome Sequencing using the Ion Torrent Personal Genome Machine (PGM). Forensic Sci. Int. Genet..

[5] Marshall C., Sturk-Andreaggi K., Daniels-Higginbotham J., Oliver R.S., Barritt-Ross S., McMahon T.P. (2017). Performance Evaluation of a Mitogenome Capture and Illumina Sequencing Protocol using Non-Probative, Case-Type Skeletal Samples: Implications for the use of a Positive Control in a Next-Generation Sequencing Procedure. Forensic Sci. Int. Genet..

[6] Strobl C., Eduardoff M., Bus M.M., Allen M., Parson W. (2018). Evaluation of the Precision ID Whole MtDNA Genome Panel for Forensic Analyses. Forensic Sci. Int. Genet..

[7] Eduardoff M., Xavier C., Strobl C., Casas-Vargas A., Parson W. (2017). Optimized mtDNA Control Region Primer Extension Capture Analysis for Forensically Relevant Samples and Highly Compromised mtDNA of Different Age and Origin. Genes.

[8] Ruiz-Pesini E., Lott M.T., Procaccio V., Poole J.C., Brandon M.C., Mishmar D., Yi C., Kreuziger J., Baldi P., Wallace D.C. (2007). An Enhanced MITOMAP with a Global mtDNA Mutational Phylogeny. Nucleic Acids Res..

[9] Zhang S. (2020). MITOMAP: Mitochondrial DNA Base Substitution Diseases: rRNA/tRNA Mutations with Cfrm Status. https://www.mitomap.org/foswiki/bin/view/MITOMAP/MutationsRNACfrm.

[10] Zhang S. (2020). MITOMAP: Mitochondrial DNA Base Substitution Diseases: Coding and Control Region Point Mutations with Cfrm Status. https://www.mitomap.org/foswiki/bin/view/MITOMAP/MutationsCodingControlCfrm.

[11] Andrews R.M., Kubacka I., Chinnery P.F., Lightowlers R.N., Turnbull D.M., Howell N. (1999). Reanalysis and Revision of the Cambridge Reference Sequence for Human Mitochondrial DNA. Nat. Genet..

[12] Gu Z., Gu L., Eils R., Schlesner M., Brors B. (2014). Circlize Implements and Enhances Circular Visualization in R. Bioinformatics.

[13] Parson W., Dur A. (2007). EMPOP—A Forensic mtDNA Database. Forensic Sci. Int. Genet..

[14] Parson W., Gusmao L., Hares D., Irwin J., Mayr W., Morling N., Pokorak E., Prinz M., Salas A., Schneider P. (2014). DNA Commission of the International Society for Forensic Genetics: Revised and Extended Guidelines for Mitochondrial DNA Typing. Forensic Sci. Int. Genet..

[15] Parson W., Bandelt H.J. (2007). Extended Guidelines for mtDNA Typing of Population Data in Forensic Science. Forensic Sci. Int. Genet..

[16] Huber N., Parson W., Dür A. (2018). Next Generation Database Search Algorithm for Forensic Mitogenome Analyses. Forensic Sci. Int. Genet..

[17] Zhou Y., Guo F., Yu J., Liu F., Zhao J., Shen H., Zhao B., Jia F., Sun Z., Song H. (2016). Strategies for Complete Mitochondrial Genome Sequencing on Ion Torrent PGM™ Platform in Forensic Sciences. Forensic Sci. Int. Genet..

[18] Ma K., Zhao X., Li H., Cao Y., Li W., Ouyang J., Xie L., Liu W. (2018). Massive Parallel Sequencing of Mitochondrial DNA Genomes from Mother-Child Pairs using the Ion Torrent Personal Genome Machine (PGM). Forensic Sci. Int. Genet..

[19] Kutanan W., Kampuansai J., Srikummool M., Kangwanpong D., Ghirotto S., Brunelli A., Stoneking M. (2017). Complete Mitochondrial Genomes of Thai and Lao Populations Indicate an Ancient Origin of Austroasiatic Groups and Demic Diffusion in the Spread of Tai-Kadai Languages. Hum. Genet..

[20] Wang M., Wang Z., He G., Wang S., Zou X., Liu J., Wang F., Ye Z., Hou Y. (2020). Whole Mitochondrial Genome Analysis of Highland Tibetan Ethnicity using Massively Parallel Sequencing. Forensic Sci. Int. Genet..

[21] Mengge W., Guanglin H., Yongdong S., Shouyu W., Xing Z., Jing L., Zheng W., Hou Y. (2020). Massively Parallel Sequencing of Mitogenome Sequences Reveals the Forensic Features and Maternal Diversity of Tai-Kadai-Speaking Hlai Islanders. Forensic Sci. Int. Genet..

[22] Ramos A., Santos C., Mateiu L., Gonzalez Mdel M., Alvarez L., Azevedo L., Amorim A., Aluja M.P. (2013). Frequency and Pattern of Heteroplasmy in the Complete Human Mitochondrial Genome. PLoS ONE.

[23] Simão F., Strobl C., Vullo C., Catelli L., Machado P., Huber N., Schnaller L., Huber G., Xavier C., Carvalho E.F. (2019). The Maternal Inheritance of Alto Paraná Revealed by Full Mitogenome Sequences. Forensic Sci. Int. Genet..

[24] Avila E., Graebin P., Chemale G., Freitas J., Kahmann A., Alho C. (2019). Full mtDNA Genome Sequencing of Brazilian Admixed Populations: A Forensic-Focused Evaluation of a MPS Application as an Alternative to Sanger Sequencing Methods. Forensic Sci. Int. Genet..

[25] Taylor C., Kiesler K., Sturk-Andreaggi K., Ring J.D., Parson W., Vallone P., Marshall C. Platinum Quality Mitogenome Haplotypes form U.S. Populations.

[26] Anderson S., Bankier A.T., Barrell B.G., de Bruijn M.H., Coulson A.R., Drouin J., Eperon I.C., Nierlich D.P., Roe B.A., Sanger F. (1981). Sequence and Organization of the Human Mitochondrial Genome. Nature.

[27] van Oven M. (2015). PhyloTree Build 17: Growing the Human Mitochondrial DNA Tree. Forensic Sci. Int. Genet. Suppl. Ser..

[28] Sturk-Andreaggi K., Peck M.A., Boysen C., Dekker P., McMahon T.P., Marshall C.K. (2017). AQME: A Forensic Mitochondrial DNA Analysis Tool for Next-Generation Sequencing Data. Forensic Sci. Int. Genet..

[29] Holland M.M., Pack E.D., McElhoe J.A. (2017). Evaluation of GeneMarker^®^ HTS for Improved Alignment of mtDNA MPS Data, Haplotype Determination, and Heteroplasmy Assessment. Forensic Sci. Int. Genet..

[30] Budowle B., Gyllensten U., Chakraborty R., Allen M. (2005). Forensic analysis of the mitochondrial coding region and association to disease. Int. J. Legal Med..

